# Makaluvamine G from the Marine Sponge *Zyzzia fuliginosa* Inhibits Muscle nAChR by Binding at the Orthosteric and Allosteric Sites

**DOI:** 10.3390/md16040109

**Published:** 2018-03-28

**Authors:** Denis S. Kudryavtsev, Ekaterina N. Spirova, Irina V. Shelukhina, Lina V. Son, Yana V. Makarova, Natalia K. Utkina, Igor E. Kasheverov, Victor I. Tsetlin

**Affiliations:** 1 Shemyakin-Ovchinnikov Institute of Bioorganic Chemistry, Russian Academy of Sciences, Miklukho-Maklaya Street, 16/10, 117997 Moscow, Russia; katya_spirova@mail.ru (E.N.S.); shelukhina.iv@yandex.ru (I.V.S.); lina.son@phystech.edu (L.V.S.); yanson1@yandex.ru (Y.V.M.); iekash@ibch.ru (I.E.K.); vits@ibch.ru (V.I.T.); 2 Moscow Institute of Physics and Technology, Institutsky Per. 9, Dolgoprudny, 141700 Moscow Region, Russia; 3 G.B. Elyakov Pacific Institute of Bioorganic Chemistry (PIBOC), Russian Academy of Sciences, Prospect 100 let Vladivostoku, 159, 690022 Vladivostok, Russia; utkinan@mail.ru

**Keywords:** marine natural products, nicotinic receptors, slow-channel, myasthenic

## Abstract

Diverse ligands of the muscle nicotinic acetylcholine receptor (nAChR) are used as muscle relaxants during surgery. Although a plethora of such molecules exists in the market, there is still a need for new drugs with rapid on/off-set, increased selectivity, and so forth. We found that pyrroloiminoquinone alkaloid Makaluvamine G (MG) inhibits several subtypes of nicotinic receptors and ionotropic γ-aminobutiric acid receptors, showing a higher affinity and moderate selectivity toward muscle nAChR. The action of MG on the latter was studied by a combination of electrophysiology, radioligand assay, fluorescent microscopy, and computer modeling. MG reveals a combination of competitive and un-competitive inhibition and caused an increase in the apparent desensitization rate of the murine muscle nAChR. Modeling ion channel kinetics provided evidence for MG binding in both orthosteric and allosteric sites. We also demonstrated that theα1 (G153S) mutant of the receptor, associated with the myasthenic syndrome, is more prone to inhibition by MG. Thus, MG appears to be a perspective hit molecule for the design of allosteric drugs targeting muscle nAChR, especially for treating slow-channel congenital myasthenic syndromes.

## 1. Introduction

Nicotinic acetylcholine receptors (nAChRs) are ligand-gated ion channels organized as pentameric complexes expressed predominantly on the plasma membrane [1]. One of the most well-studied members of this group is muscle nAChR localized specifically at the end-plate of the neuromuscular synapse. Its main function is the transmission of signals from motor neurons to muscle fibers. Due to its defined position on the skeletal muscles, this nAChR represents a valuable therapeutic target. Muscle nAChR blockers have been used for a long time as myorelaxants [2]. Besides that, antagonists of this receptor, among which are peptides (α-conotoxins) and proteins (α-bungarotoxin and other snake venom α-neurotoxins), have a wide range of applications as research tools in structural neurochemistry [3]. Classic muscle relaxants bind at the same site as acetylcholine. They either cause uncontrolled muscle nAChR activation followed by the desensitization of the receptor and muscle relaxation (as is the case for depolarizing myorelaxant succinylcholine [4]) or they block acetylcholine binding and prevent the signal transmission through the end-plate (curaremimetics and other non-depolarizing myorelaxants). Succinylcholine is usually used during intubation and electroconvulsive therapy, while curaremimetics are used during surgical operations as a complementary to anesthetics [5].

Acetylcholine, an endogenous agonist of muscle nAChRs, binds at the interface between the extracellular domains of the receptor subunits, while the ion channel of the receptor is composed of the transmembrane segments of the subunits. Therefore, some path for information transmission between the distant parts of the protein complex (extracellular and transmembrane domains) should exist. Muscle nAChR activity is based on the allosteric transitions between different functional states [6]. One such transition is the gating process during which muscle nAChR with the “closed” ion channel upon acetylcholine binding transforms to the “open” state. Another interesting transition underlies the desensitization—the transformation of the “open” acetylcholine-bound state to the “closed” acetylcholine-bound state. The desensitized state of the nAChR is already acetylcholine-bound, thus, it cannot react to the external acetylcholine and the ion channel remains closed despite the presence of an agonist at the synaptic cleft. The deregulation of the desensitization as a result of certain point gain-of-function mutations leads to the slow-channel congenital myasthenic syndrome (SCCMS), a very rare condition against which no specific treatment has been developed [7]. 

In principle, antagonists or channel blockers can be used to treat “excessively active” receptors. Most muscle nAChR antagonists work as strong muscle relaxants suppressing the muscle tonus. Application of classic muscle relaxants requires skilled anesthesiology personnel and could hardly ever be used in therapy under “slow-channel” myasthenic conditions. However, there are two therapeutics which are used for such purposes now, namely quinidine [8,9] and fluoxetine [10]. They act as non-competitive blockers of nAChRs, but their binding site has not been yet identified [11,12]. Both molecules were not initially dedicated to slow-channel conditions therapy and have many undesirable side-effects. Thus, the design and search for new muscle nAChR ligands for treating SCCMS remain important tasks. That is why compounds acting on muscle nAChRs draw our attention and one of them, Makaluvamine G (MG), showed interesting properties.

**Figure 1:**
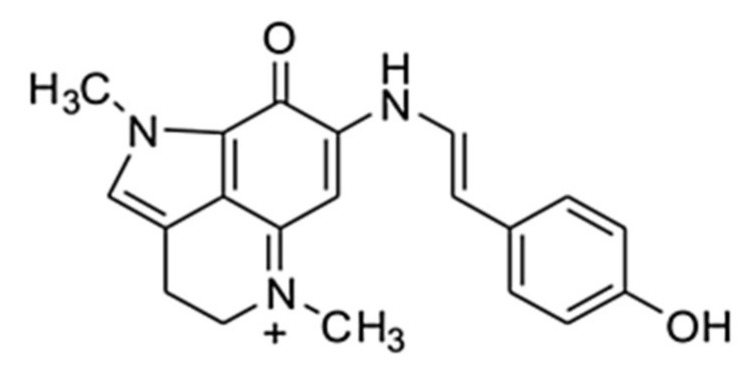
The Makaluvamine G (MG) structure.

MG (Figure 1) was isolated for the first time from an Indonesian marine *Zyzzia* sponge of *Histodermella* genus and showed moderate (IC_50_ ~ 3 μM) topoisomerase I inhibiting, immunomodulatory and cytotoxic activities at concentrations ranging from about 1 μM to about 70 μM [13].

We have recently discovered that MG inhibits murine muscle nAChR and competes with ^125^I-α-bungarotoxin at the orthosteric binding site of muscle-type nAChR of the *Torpedo californica* electric organ [14]. A more detailed analysis of muscle nAChR inhibition by MG and molecular modeling studies of its binding at the muscle nAChR orthosteric site are the purposes of the current study.

## 2. Results and Discussion

### 2.1. Makaluvamine G Shows Properties of an Un-Competitive Blocker at theMuscle nAChR

**Figure 2:**
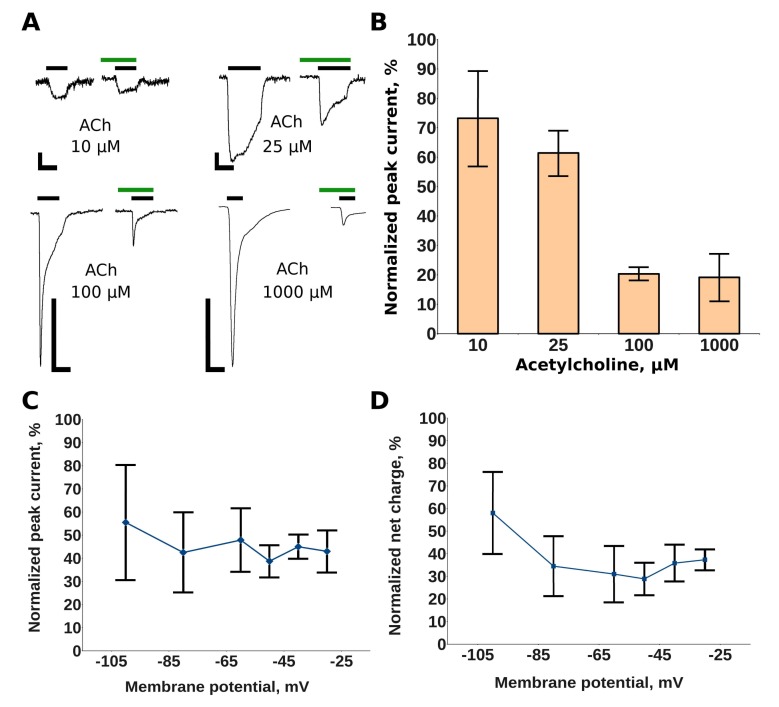
The electrophysiological study of MG action on muscle nicotinic acetylcholine receptor (nAChR). (**A**) Representative traces of acetylcholine-evoked current in control conditions (black bars) and in the presence of 2.5 μM MG (green bars). Measurements were acquired at a holding potential of −70 mV. The current scale bars shown are 100 nA, 250 nA, 500 nA, and 1 μA, respectively. The time scale bars are 10 s in all cases; (**B**) the current amplitude of responses to various acetylcholine (ACh) concentrations in the presence of 2.5 μM MG normalized to the amplitude of the control responses; (**C**) the dependence of ACh-evoked current inhibition by 2.5 μM MG on the membrane potential in terms of the current amplitude. The acetylcholine concentration used was30 μM; (**D**) the dependence of ACh-evoked current inhibition by 2.5 μM MG on the membrane potential in terms of the net charge. The acetylcholine concentration used was 30 μM. On (**B**–**D**) the data are plotted as mean values with 95% confidence intervals.

MG at 2.5 μM co-applied with acetylcholine to the murine muscle nAChR expressed in *Xenopus* oocytes reduced the acetylcholine-evoked current differently, depending on the application of 10, 25, 100, or 1000 μM acetylcholine (Figure 2A). In terms of peak current amplitude, the inhibition extent ranged from 27% at 10 μM acetylcholine to 81% at 1000 μM (Figure 2B). Un-competitive (not to be confused with non-competitive, which does not correlate positively with agonist concentration) inhibition could arise from the direct open-channel blocking, as it is known for memantine blocking of the *N*-methyl-d-aspartate (NMDA) receptor [15], as well as from the receptor modulation, as described for muscle nAChR interaction with 2,6-dimethylaniline [16]. In this case, the antagonist can bind to some allosteric site and change the kinetic properties of the ion channel. Indeed, if some molecule has an affinity for the acetylcholine-bound receptor, it would have a greater probability to block the channel’s pore if more receptors are activated on the membrane (at higher acetylcholine concentrations). Alternatively, the antagonist molecule might form a complex with acetylcholine itself. If such a hypothetical complex would have a greater affinity toward the receptor, such interactions could explain the results shown in Figure 2B. A possibility of the combination of different mechanisms (including “classic” orthosteric inhibition) should also be kept in mind.

### 2.2. Makaluvamine G Does Not Have Channel Blocker Activity

To assess the possibility of an open-channel block, we performed electrophysiological experiments using membrane potentials in the range from –30 to –100 mV. To avoid the possible influence of acute receptor desensitization, we calculated both the amplitude reduction of acetylcholine (ACh)-evoked current (Figure 2C) and the ACh-evoked net-charge flow through the channel (Figure 2D). In this case, we took into account the possible reduction of the peak width with no amplitude change. No increase in the inhibition of the receptor activity at higher negative membrane potentials (which would be interpreted as a sign of a channel block) was detected. This could mean that a direct channel block is not consistent with our data and that other explanations of un-competitive receptor inhibition should be found.

### 2.3. Makaluvamine G Binds at the Orthosteric Sites of the Muscle-Type nAChR

**Figure 3:**
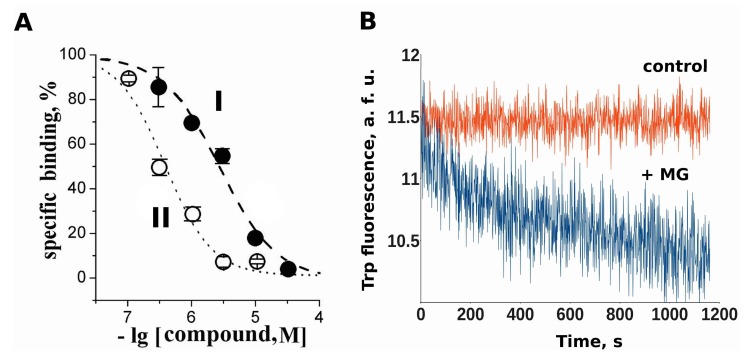

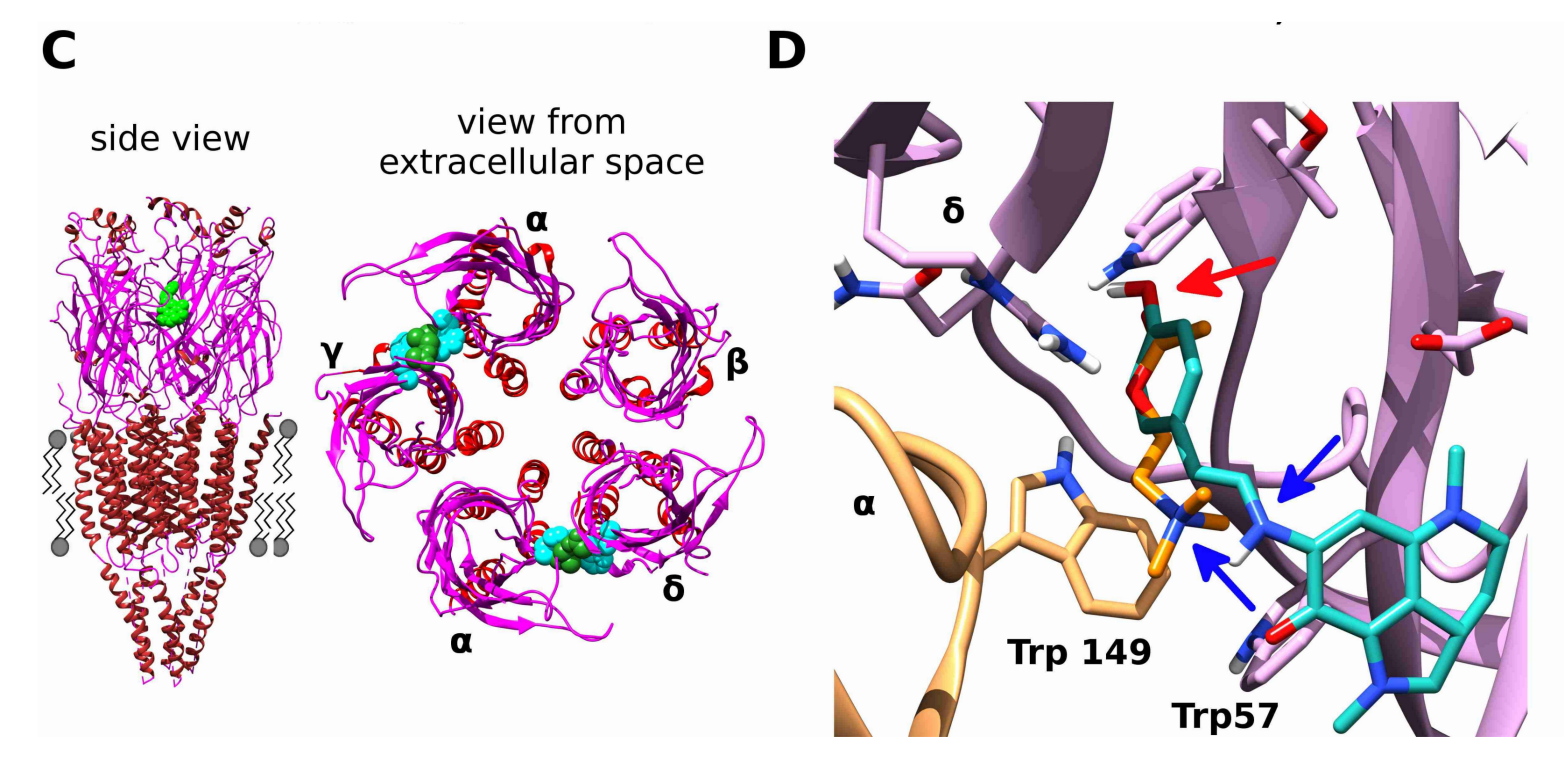
The binding site determination. (**A**) The inhibition of the initial rate of [^125^I]-αBgt binding to the *Torpedo californica* nAChR with MG (I) and *d*-tubocurarine (II). Each point has a mean ± standard error of the mean value of two or three measurements for each concentration. The curves were fitted by the ORIGIN 7.5 program using the Dose Response model. The respective IC_50_ values were 2.8 ± 0.3 μM and 0.37 ± 0.04 μM; **(B**) the intrinsic *T. californica* muscle-type nAChR Trp fluorescence quenching by MG. The binding of MG to this receptor decreases the fluorescence of the Trp residues at the binding sites; (**C**) the schematic representation of MG docking to orthosteric binding sites between the α and δ/ε subunits (side view on the left and top view on the right). MG is shown in green and the Trp residues in orthosteric sites are shown in cyan; (**D**) the superposition of acetylcholine and MG docked to the orthosteric α^+^/δ^−^ site. The positions of oxygen and nitrogen atoms similar to acetylcholine and MG are pointed by the red and blue arrows, respectively.

MG completely inhibits the radiolabeled α-Bgt binding to the *Torpedo californica* nAChR with micromolar affinity (Figure 3A; IC_50_ = 2.8 ± 0.3 μM). Complementary to that, we investigated the MG binding to the same receptor by using the intrinsic tryptophan fluorescence quenching method (λ_ex_ = 280 nm, λ_em_ = 340 nm). In good agreement with the radioligand competition data, it was found that MG induces a drop of intrinsic tryptophan fluorescence and this drop is prevented by nAChR pre-incubation with α-Bgt (Figure 3B). For the sake of data representational consistency, additional fluorescence from the α-Bgt Trp was subtracted from the data. The binding of the α-Bgt also quenched the *Torpedo* nAChR Trp fluorescence, thus, the baseline fluorescence was corrected on the figure. There are two Trp residues in each *Torpedo californica* muscle-type nAChR orthosteric sites known to be involved in α-Bgt binding: W149 of the α1 subunit (principal side) and W57 of the δ subunit or W54 of the γ subunit, located in the vicinity of the acetylcholine binding region (Figure 3C).

### 2.4. Docking of Acetylcholine and Makaluvamine G witha Model of the Α^+^Δ^−^ Subunit Interface of *Torpedo californica* Muscle-Type nAChR

Muscle-type nAChR from *T. californica* and adult murine muscle nAChR each contain two agonist binding sites: α^+^γ^−^ and α^+^δ^−^ (*Torpedo*) and α^+^δ^−^ and α^+^ε^−^ (murine), respectively (plus meaning the principal side and minus meaning the complementary side). We performed molecular docking simulations of MG binding to the model of murine nAChR α^+^δ^−^ site because it is present in both these nAChR subtypes used in our experiments. The docking of the MG molecule with the α^+^δ^−^ inter-subunit site of the muscle-type nAChR resulted in only four possible binding positions. The most populated cluster of docking results (which enclosed 1858 out of 2000 dockings) is represented in Figure 3D. Note that the tyramine moiety of MG occupies the binding site in a manner somewhat similar to that of ACh: the carbonyl oxygen of acetylcholine takes the position close to that of hydroxyl oxygen of Makaluvamine G, while the Makaluvamine’s secondary amine forms close contacts with Trp149, similarly to what the quaternary amine of acetylcholinedoes.

### 2.5. Makaluvamine G Is More Effective against the Gain-Of-Function Muscle nAChRMutant Α1(G153S) Than against the Wild-Type Receptor

**Figure 4:**
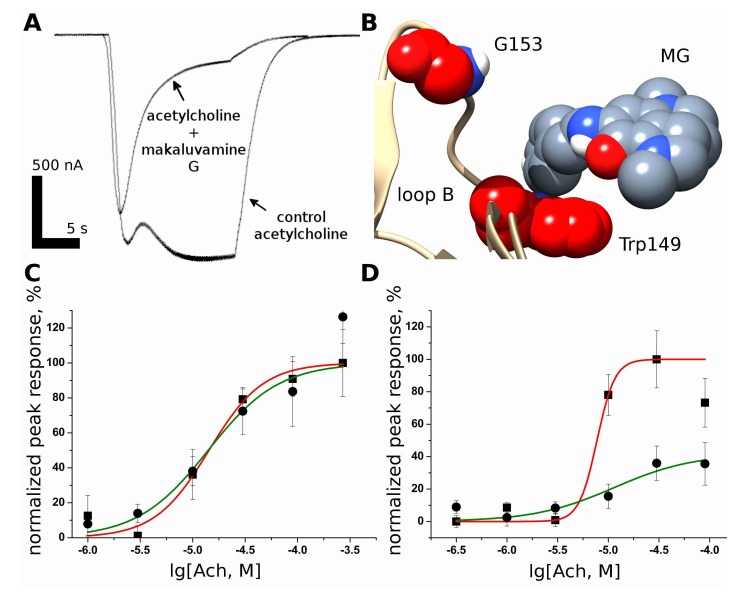
Receptor mutagenesis and fluorescence assay. (**A**) Comparison of acetylcholine-evoked current time course in control conditions and under co-application of MG; (**B**) the putative structure of the MG complex with muscle nAChR. Note that the G153 residue (involved in the receptor gating according to the literature data) is situated in loop B near the orthosteric site. Loop C residues are not shown; (**C**) the dose-response of the wild-type (WT) muscle nAChR to acetylcholine is not significantly altered by 2.5 μM MG according to the induced changes in the cytoplasmic Ca^2+^ level. The curve equation is % response = 100/{1 + ([compound]/EC_50_)*^n^*}, where EC_50_ is the half-maximal response and *n* is the Hill coefficient. The control curve is shown in red, the curve showing data of the MG is shown in green; (**D**) the gain-of-function point mutation G153S in the α1 subunit which increases the receptor sensitivity to ACh and decreases the acute desensitization rate also increases the receptor sensitivity to MG. The dose-response of the mutant G153S muscle nAChR to the acetylcholine insignificantly altered by 2.5 μM MG according to the induced changes in the cytoplasmic Ca^2+^ level. The control curve is shown in red, the curve showing the data with the MG is shown in green. These data suggest that there is a close interconnection between the muscle nAChR desensitization and inhibition of this receptor by MG. We found that 2.5 μM MG does not affect the WT receptor dose response to acetylcholine measured by this method, but it is sufficient to significantly (*p* < 0.01, *t*-test for normalized maximal responses) suppress the acetylcholine-evoked Ca^2+^ concentration rise mediated by the G153S mutant (Figure 4C,D, respectively).

The MG inhibitory action tends to grow with an increase in the acetylcholine concentration. Thus, the higher the fraction of nAChR activated, the larger the number of receptors that are functionally blocked (Figure 2A,B). On the other hand, co-application of MG with acetylcholine increases the apparent rate of muscle nAChR desensitization (Figure 4A). These properties could be beneficial in some cases of muscle nAChR gain-of-function mutations. One might expect that those nAChRs which are more prone to acetylcholine-induced activation, such as the slow-channel congenital myasthenic syndrome (SCCMS) associated gain-of-function mutant, could be “corrected” back to the normal activity level by MG.

To check this concept, we engineered muscle murine nAChR containing the α1[G153S] gain-of-function mutation found in patients with SCCMS. The Gly153 residue is situated in the loop B near the conservative Trp149 residue crucial for nAChR activation [17] (Figure 4B). It also has been shown that G153 residue is involved in the receptor gating [18].

Since severe muscle weakness of affected persons is attributed to Ca^2+^ accumulation in the endplates [19], we decided to test the effectiveness MG via Ca^2+^ detection in the fluorescence experiments (Figure 4C,D) using the method described in reference [20].

Thus, it can be concluded that MG inhibition of muscle nAChR could be utilized in counterbalancing the effects of the gain-of-function mutation and, at least under certain conditions, could discriminate between wild-type (WT) and mutant receptors. This is of particular interest because SCCMS mutations are usually dominant and both WT and mutant receptors could be expressed in affected heterozygous individuals.

### 2.6. Makaluvamine G Mode of Action Is Due to the Distinct Channel States

**Figure 5:**
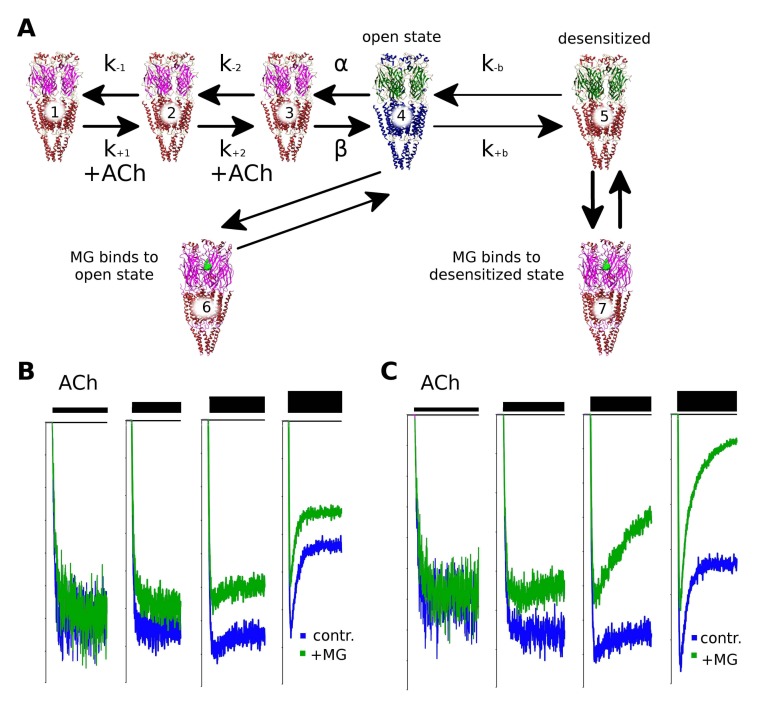
(**A**) The kinetic scheme of muscle nAChR activation and desensitization (states from 1 to 5) and the proposed states of the receptor that correspond to the un-competitive mode of inhibition (state 6) and the apparent increase of desensitization (state 7). The states with transmembrane segments depicted in red are non-conducting, the only conducting state is shown in green and blue, and the desensitized state is shown in green and red; (**B**) the currents simulated according to the kinetic scheme, MG binding only to the open state is considered. Note the increase of inhibition (but not the desensitization rate) with the increase of the ACh concentration (control simulated currents are shown in blue, simulated currents in the presence of MG are shown in green).The currents were scaled to match the maximal response amplitude; (**C**) the currents simulated according to the kinetic scheme, MG binding to both open and desensitized states is considered. Note that the apparent desensitization rate increased (simulated current in the presence of MG decays faster). The control simulated currents are shown in blue, the simulated currents in the presence of MG are shown green, the currents were scaled to match the maximal response amplitude. Thicker bars designate higher acetylcholine concentrations.

To shed light on the mechanism of nAChR inhibition, we constructed a kinetic model of the receptor’s ion channel activation and inactivation based on the literature data [21] and applied different scenarios of possible MG action (Figure 5). The receptor activation was modeled using the QUB Express software [22] which utilizes Markov chains to explore the dynamics of ligand-gated or voltage-gated ion channel activation. The basic scheme of the muscle nAChR channel states is shown in the top line of Figure 5a. The un-liganded closed state **1** binds acetylcholine with thek_+1_ constant, then the monoliganded state **2** binds the second acetylcholine molecule with the constant k_+2_. The resulting di-liganded state **3** spontaneously converts to the open state **4** with a constant conventionally named β (reverse conversion from open to closed di-liganded state **3** is characterized by the so-called α constant). The desensitization of the nAChR could be explained by the conversion of the open state to the di-liganded desensitized state **5** with the k_+b_ constant (constant of the reverse reaction is denoted as k_−b_). We hypothesized that MG binds to some of the receptor states and modifies the kinetic scheme in such a way that un-competitive inhibition occurs and the apparent desensitization rate increases.

Interestingly, we found that the modeling of the MG competition with acetylcholine for the orthosteric sites (binding of MG to the closed **1** and **2** states) cannot reproduce both the un-competitive inhibition and the apparent desensitization increase (not shown). Only a combination of mechanisms could explain unusual features of MG inhibition of muscle nAChR. Binding to the open state of the receptor (the state which bound two acetylcholine molecules) converts it to the closed state **6** which corresponds to the increase of inhibition depending on the acetylcholine concentration: the higher the acetylcholine concentration that is applied, the more pronounced the inhibition that could be observed (Figure 5B). However, binding to the open state does not reproduce the increase in the desensitization rate (that is, does not sharpen the peak of the agonist-evoked current) that is observed in experiments (Figure 4A). Only the addition of MG binding to the desensitized state on the kinetic scheme reproduces the increased desensitization rate (equilibrium between states **5** and **7**, Figure 5C). States **6** and **7** are bound to acetylcholine which infers MG binding to some allosteric site(s). Indeed, if the combination of the un-competitive mode of action and the apparent increase in the desensitization rate are mediated by the two states of the receptor which already have acetylcholine molecules in the orthosteric site, the only feasible explanation is that of MG binding outside the orthosteric sites (that is, at the allosteric sites). This mechanism complements the orthosteric binding detected by the radioligand competition test.

Thus, Makaluvamine’s G un-competitive mode of inhibition and desensitization-increasing property could be explained by the additional binding sites for this ligand on muscle nAChR. A more thorough investigation of these hypothetical binding sites is in our future plans.

### 2.7. Makaluvamine G at Higher Micromolar Concentration Inhibits the Α4β2 nAChR

**Figure 6:**
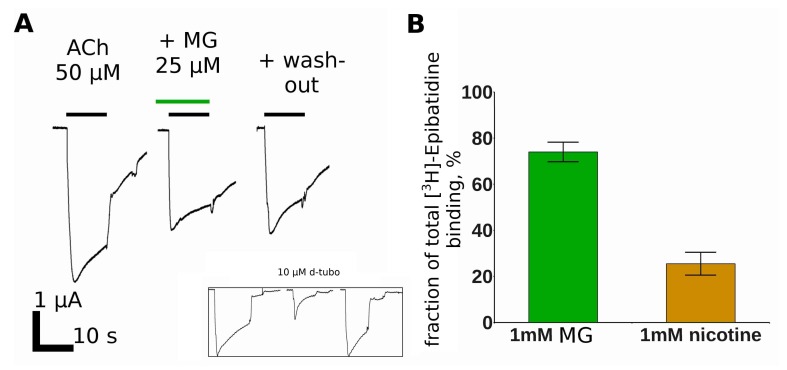
The Makaluvamine G interaction with theα4β2 nAChR. (**A**) The inhibition of the ACh-evoked currents in the *Xenopus* oocytes expressing rat α4β2 nAChR. Acetylcholine (50 μM) and MG (25 μM) applications are shown by the black and green bars, respectively. The inset picture shows the effect of 10 μM d-tubocurarine on this receptor; (**B**) The inhibition of equilibrium [^3^H]-epibatidine binding to the transfected SH-EP cells expressing α4β2 nAChR by 1 mM of MG compared to 1 mM of nicotine. The data are presented as the mean ± standard error.

MG appears promising for the design of drugs to treat SCCMS. However, it is also essential to know other possible targets of this compound. In our previous work, it was found that the MG is at least one order of magnitude more potent at the muscle than at the human α7 nAChR, and does not inhibit homopentameric α1 GlyR [14]. We decided to check if MG interacts with such an abundant receptor as the heteromeric rat α4β2 nAChR. The electrophysiological experiments revealed only weak inhibition of this receptor by MG at a high micromolar range of concentrations (Figure 6A). It is worth noting that MG could be washed out more slowly than d-tubocurarine (Figure 6A, inset), but the inhibition was clearly reversible and the normal current amplitude recovered in 15–20 min (data not shown).

The radioligand competition assay showed that MG inhibits [^3^H]-epibatidine binding to some extent. However, the inhibition was partial even at test ligand concentrations up to 1 mM. The apparent disagreement between the electrophysiology and radioligand competition test could be attributed to the difference between the expression systems used in each method (*Xenopus* oocytes and SH-EP cell line, respectively). Another possible explanation—the inhibition of theα4β2 nAChR through allosteric sites which could not be detected by the orthosteric radioligand ([^3^H]-epibatidine) competition. It has already been mentioned that MG shows cytotoxicity at 1–70 μM on different types of cells [13]. Thus, some effects could be attributed to the non-specific membrane interaction. However, no obvious membrane integrity disruption was detected during the time course of a typical electrophysiological experiment. Regardless, these results show that MG inhibits muscle nAChR much more potently than α4β2 nAChR.

### 2.8. Makaluvamine G at Higher Concentrations Inhibits GABA_A_R

Several nAChR ligands possess an affinity toward γ-aminobutiric acid receptors (GABA_A_R) and vice versa: *d*-tubocurarine and α-cobratoxin act on GABA_A_R with sub-micromolar affinity [23], bicuculline inhibits α9 nAChR [24], and even GABA itself activates muscle nAChR [25]. Therefore, testing MG action on GABA_A_R was an essential part of this study.

**Figure 7:**
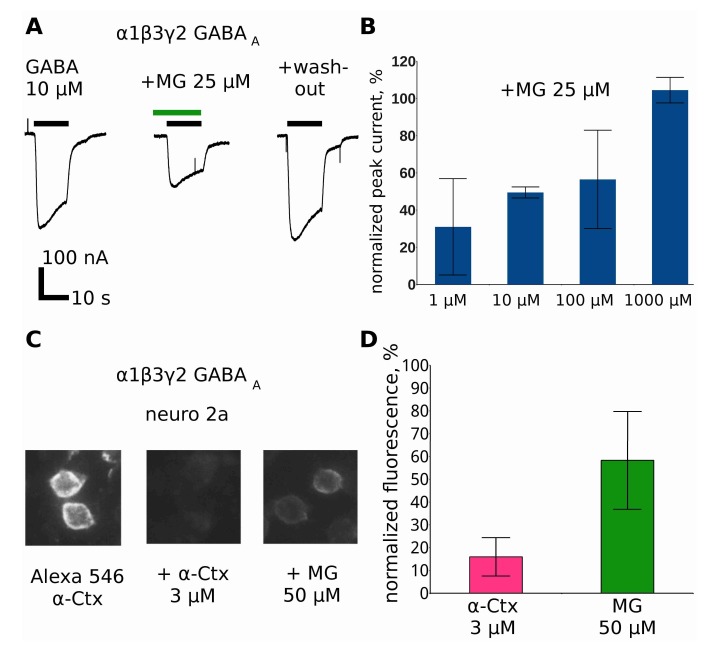
(**A**) MG at 25 μM suppresses GABA-evoked currents in *Xenopus* oocytes expressing α1β3γ2 GABA_A_R. GABA (10 μM) and MG (25 μM) applications are shown by the black and green bar, respectively. (**B**) The normalized GABA-evoked current in the presence of 25 μM MG. The GABA concentration varied from 1 μM to 1 mM. Note that, in contrast to the muscle nAChR, the inhibition is surmounted by high agonist concentration. The bars represent the mean value ± 95% confidence interval. (**C**) MG inhibits the fluorescent α-Ctx binding to Neuro2a cells overexpressing GABA_A_R. (**D**) Only partial inhibition of the fluorescent α-Ctx binding to GABA_A_R could be achieved at 50 μM MG, showing that MG acts at the orthosteric site but with a lower affinity compared to the muscle nAChR. The bars represent the mean value ± 95% confidence interval.

Indeed, we found that MG at a concentration of 25 μM inhibits GABA_A_R (Figure 7A). Unlike muscle nAChR, GABA_A_R is inhibited in a surmountable way—the inhibition effect could be suppressed by higher GABA concentrations (Figure 7B).

In good agreement with the surmountable inhibition, MG shows the ability to displace fluorescent α-cobratoxin derivative binding to cells overexpressing GABA_A_R (Figure 6C,D).

### 2.9. Discussion

Previously we reported that MG competes with α-bungarotoxin at the muscle-type nAChR, thus, indicating binding to the orthosteric sites [14]. The co-application of MG with the agonist acetylcholine in electrophysiology tests revealed its inhibiting action with the inhibition constant comparable with that of d-tubocurarine. Interestingly, in the first report on MG [13], it was pointed out that this compound was not toxic to mice at doses up to 210 mg/kg, which corresponds to 629 μmoles/kg. That is quite a high dose comparing to theLD_50_ of d-tubocurarine which is less than 0.7 μmole/kg [26]. A possible explanation of such a low in vivo toxicity could lie in the molecular mechanism of the MG action. Here we found that the inhibition of the peak current is dependent on the acetylcholine concentration and that it is more than two-fold higher at the saturating concentrations of acetylcholine (Figure 2B). This observation can explain why MG does not block respiratory muscles despite being active against the muscle nAChR at low micromolar concentrations. Further experiments showed that there is no significant dependence of the inhibition on the membrane potential (Figure 2C,D) and we could exclude a mechanism based on the direct block of the ion channel.

MG inhibits ^125^I-α-bungarotoxin binding to the muscle-type nAChR of *Torpedo californica*, thus, indicating that it binds at the orthosteric site. We performed molecular docking to the model of muscle nAChR to explore the possible modes of MG binding. To validate our docking procedure, the docking of the acetylcholine molecule to the molecular model of an α^+^δ^−^ inter-subunit site of the muscle-type nAChR was conducted. The α^+^δ^−^ site was chosen because it is present in both the fetal and adult forms of the muscle nAChR. The acetylcholine docking resulted in structures that are in a good agreement with the published experimental data showing a similar acetylcholine position in the binding site of AChBP (PDB 3WIP) [27]. Docking of the MG molecule to the α^+^δ^−^ inter-subunit site of muscle-type nAChR revealed a possible binding position that shares some features with acetylcholine docked at the same site. For example, the very similar positions of oxygen and nitrogen atoms (Figure 3D, pointed by arrows) in the vicinity of the Trp149 residue that is known to be crucial for the action of nAChR agonists [17]. Moreover, the docking identified that the pyrroloiminoquinone moiety of MG forms hydrophobic contacts with several other residues of the receptor, among them is the Tyr190 in α1 subunit and Asp59 residue in the complementary (in this case δ) subunit. Mutations of these residues have earlier been shown to influence gating mechanisms of the receptor by altering its activity [28,29]. Thus, according to docking results, MG molecule might form contacts with the receptor residues that are involved in receptor gating and desensitization while binding at the orthosteric site.

However, we noticed an increase in the acute desensitization rate of the muscle nAChR upon the MG application (Figure 4A). Moreover, Trp149 of the orthosteric site is located in the loop B (Figure 4B) where its function is affected by the conservative residue Gly153 [17]. The mutation of the latter to Ser153 produces a receptor with increased affinity to acetylcholine [18]. In other words, the effect of theG153S mutation is somewhat opposite to the effect of MG. We engineered a mutant α1(G153S)β1δε muscle nAChR and analyzed the MG activity in comparison to the wild-type receptor (Figure 4C,D). Fluorescent detection of cytoplasmic calcium was chosen as a method for activity detection because calcium is known to be a secondary messenger and because some of the negative effects of the gain-of-function mutations (such as G153S) are thought to be mediated by increased calcium concentration in the affected muscle fibers [19]. Interestingly, we found that MG is more active against the α1(G153S)β1δε muscle nAChR (Figure 4C,D). These data suggest a possible utility of MG as a hit compound in the rational design of new drugs against SCCMS and other conditions related to gain-of-function mutations of nAChRs. It should be noted that Figure 4D reveals a mode of inhibition different from the competitive one: the maximal response of the receptor is reduced by 42 ± 13% and the acetylcholine EC_50_ value is shifted from 0.27 (0.19–0.39) μM to 11.34 (4.86–26.47) μM. This result is in good agreement with the electrophysiological data shown in Figure 2A,B, where the inhibition of the receptor by MG could not be surmounted by the increasing acetylcholine concentration. Such a mode of inhibition suggests the existence of another MG binding site or sites on the muscle nAChR.

To investigate the existence of other binding sites on the muscle nAChR, modeling of the kinetic states of the receptor was performed using Markov chains the QUB Express software (Figure 5). It is obvious that the muscle nAChR inhibition by MG in part occurs via competition with the agonist (acetylcholine) for the orthosteric binding site. However, such competition does not explain the un-competitive mode of inhibition identified by the electrophysiology experiments and supported by the fluorescent calcium detection. The addition of the hypothetical closed channel state **6** (see Figure 5A), which is generated upon MG binding to the open state **4**, reproduces the observation of un-competitive inhibition: the computer-simulated current is inhibited more in the case of higher acetylcholine concentrations (compare Figure 5B with Figure 2A). The increase in the apparent acute desensitization (Figure 4A) is not reproduced by the addition of only state **4**. Hypothetical state **7** is needed to observe the increase in the desensitization in the kinetic model (Figure 5C). Thus, a combination of the orthosteric and allosteric binding seems to be needed to explain all the muscle nAChR-inhibiting properties of MG. However, further studies (for example, single channel electrophysiology combined with site-directed mutagenesis) are required to be donein order to decipher the particular molecular mechanism of MG at the muscle nAChR.

Earlier it was found that apart from the muscle nAChR, MG binds to the *Lymnaea stagnalis* acetylcholine-binding protein, α7 nAChR, and does not inhibit the human α1 glycine receptor [14]. In the present study, we report MG binding and functional inhibition of α4β2 nAChR (Figure 6) and α1β3γ2 GABA_A_R (Figure 7). In both cases, the inhibition is weaker than that of the muscle nAChR and only partial inhibition could be achieved with 25 μM MG.

In summary, Makaluvamine G might present a potentially useful hit molecule for the rational design of drugs acting like un-competitive antagonists of nAChRs and other Cys-loop receptors. This might be of special interest in the light of findings that endogenous nAChR ligands from the Ly6 family show some properties similar to un-competitive inhibitors [30,31,32]. Thus, small molecules mimicking properties of endogenous modulators of Cys-loop receptors could be of a high value.

## 3. Experimental Section

*Two-electrode voltage clamp*. Oocytes of mature *Xenopus* frogs were removed surgically and treated with type I collagenase (Thermo Fisher Scientific, Waltham, MA, USA) dissolved in a Ca^2+^-free ND96 buffer (5 mM HEPES, 2 mM MgCl_2_, 2 mM KCl, and 96 mM NaCl; pH 7.5). After separation, μM oocytes were transferred to regular ND96 (5 mM HEPES, 2 mM MgCl_2_, 1.8 mM CaCl_2_, 2 mM KCl, and 96 mM NaCl; pH 7.5) and injected with 1–5 ng of plasmid DNA containing murine muscle nAChR α1, β1, δ, and ε subunit genes (pRBG4 vector); murine α1, β3, and γ2 GABA_A_R subunits (PCI vector); or rat α4 and β2 nAChR subunits (pcDNA 3.1 vector). Recordings were performed 24–72 h after injection. A Turbo TEC-03X amplifier (NPI electronic, Tamm, Germany) was used along with the WinWCP software.

*Homology modeling and molecular docking*. Homology models of α1 and δ subunits of the *Torpedo californica* muscle-type nAChR were obtained via the SWISS MODEL web service [33]. The acetylcholine structure was downloaded from the ZINC database [34]. The Makaluvamine G structure was constructed in Avogadro [35] and partial charges were assigned to the atoms in UCSF Chimera via theAM-BCC1 forcefield plugin [36].

*Cell cultures and transfection*. Mouse neuroblastoma Neuro2a cells or SH-EP cells were cultured in Dulbecco’s modified Eagle’s medium (DMEM, PanEco, Moscow, Russia) supplemented with 10% FBS (PAA Laboratories, Pasching, Austria). Neuro2a cells were sub-cultured for 24 h before transfection and plated at a density of 10,000 cells per well (black 96-well plate, Corning Inc, New York, NY, USA), followed by a lipofectamine (Invitrogen, Carlsbad, CA, USA)-mediated transient co-transfection of plasmid coding mouse muscle WT or mutant mouse muscle α1 (WT or mutant), β1, δ, and ε nAChR subunits (pRBG4 vector) and a fluorescent calcium sensor Case12 (pCase12-cyto vector, Evrogen, Moscow, Russia). Murine α1, β3, and γ2 GABA_A_R-lab-pCI plasmid constructs were expressed similarly, but without a sensor Case12. SH-EP cells were plated at a density of 10,000 cells per cell culture flasks (25 cm^2^, Corning) 24 h before the transfection with plasmids encoding rat α4 and β2 nAChR subunits (pcDNA 3.1 vector).

*Intrinsic tryptophane fluorescence detection*. Electric organ membranes of *Torpedo californica* at the final concentration of 1 nM of toxin-binding sites in a 20 mM Tris-HCl buffer were mixed with Makaluvamine G (final concentration 2 μM) and the control membranes were pre-incubated with 1 μM αBgt for 30 min. Measurements were done on a Cary Eclipse (Agilent, Santa Clara, CA, USA) spectrofluorimeter with a standard kinetics software (version 1.2) with excitation wavelength 280 nm and emission wavelength 340 nm (both with 5 nm slits). The data were exported to csv files and analyzed in the LibreOffice 6.0 Calc software (The Document Foundation, Berlin, Germany).

*Radioligand competition assay*. In the competition experiments with [^125^I]-αBgt, makulavamine G and *d*-tubocurarine (at varied concentrations) were pre-incubated for 3 h at room temperature with *Torpedo californica* electric organ membranes (final concentration 1.25 nM of toxin-binding sites) in 50 μL of binding buffer (20 mM Tris-HCl buffer, 1 mg/mL of bovine serum albumin, pH 8.0). After that, [^125^I]-αBgt was added to the membranes to a final concentration of 0.1 nM and the mixtures were additionally incubated for 5 min. The binding was stopped by rapid filtration on GF/C filters (Whatman) pre-soaked in 0.25% polyethylenimine, the unbound radioactivity having been removed from the filters by washout (3 × 3 mL) with a binding buffer. Non-specific binding was determined in all cases using 3 h pre-incubation with 30 μM α-cobratoxin. The results were analyzed using ORIGIN 7.5 (OriginLab Corporation, Northampton, MA, USA) fitting to a one-site dose-response curve by Equation: % response = 100/{1 + ([compound]/IC_50_)*^n^*}, where IC_50_ is the concentration at which 50% of the binding sites are inhibited and *n* is the Hill coefficient.

*Fluorescent ligand competition assay*. Neuro2a cells transiently expressing α1β3γ2 GABA_A_R were washed with the external buffer (20 mM HEPES, 140 mM NaCl, 2.8 mM CaCl_2_, 1 mM MgCl_2_, 10 mM glucose; pH 7.4). Cells were then pre-incubated with 50 μM Makulavamine G for 15 min. Then, 50 μL of 100 nM Alexa Fluor 546 α-Ctx conjugate (to make the final volume of 100 μL) was added to the cells for 20 min. To estimate the level of non-specific fluorescence intensity, simultaneous experiments with 3 μM α-Ctx was performed. Afterward, the cells were washed three times with a two-fold excess of extracellular solution. By epifluorescent microscope IX71 (Olympus, Tokyo, Japan) three fields in each experimental plate well were chosen in bright field illumination in an unbiased manner and pictures were taken with an appropriate filter combination. The fluorescent image analysis was done on 8-bit gray value pictures in TIFF format [37] and ImageJ [38] open source software. Fluorescence intensity was normalized to the mean integral intensity of the field incubated inthe presence of 50 nM Alexa Fluor 546 α-Ctx conjugate. Each experimental point is an average of the integral intensity independently measured for 12 independent fields ± Standard Deviation.

*Fluorescent detection of cytoplasmic Ca^2+^ rise*. Calcium imaging was performed following Reference [20]. Briefly, the cell medium was removed and the cells were washed with the external buffer. Then 2.5 μM MG or the vehicle was added to each well in final volumes of 50 µL for 10 min before agonist application. Acetylcholine was added to each well at final concentrations ranging from 270 µM to 300 nM. Cells were excited at 485 nm and the emitted fluorescence was detected at 535 ± 10 nm, using a multimodal microplate reader Hidex Sense (Hidex, Turku, Finland). The fluorescence was recorded every 2 s for 3 min following agonist addition. The responses were measured as peak intensity minus basal fluorescence level and were expressed as a percentage of a maximal response to the agonist. Data files were analyzed using the HidexSence software (Hidex, Turku, Finland) and the ORIGIN 7.5 software (OriginLab Corporation, Northampton, MA, USA) fitting to a one-site dose-response curve: % response = 100/{1 + ([compound]/EC_50_)*^n^*}, where EC_50_ is the half-maximal response and *n* is the Hill coefficient. Negative controls were run in the presence of 5 μM α-Ctx.

*Modeling of ion channel kinetics*. Ion channels were modeled in the QUB Express software [22]. Constants of reactions were gathered from reference [21] for the Ca^2+^-containing solution at 22 °C. Possibilities of MG binding to different states of the receptor (from 1 to 5) were explored. Each possibility was studied with four increasing simulated acetylcholine concentrations. We searched the models which would reproduce the following features of the MG muscle nAChR inhibition: un-competitive mode of inhibition (Figure 2A) and an increasing of the apparent desensitization rate (Figure 4A). Binding only to the states **4** (open di-liganded) or **5** (desensitized) and not the other states can explain un-competitiveness or increased desensitization rate (see Figure 5B for the un-competitive inhibition modeling results). Thus, a combination of both mechanisms was proposed and tested (Figure 5C).

*Site-directed mutagenesis*. Mutation G153S in mouse α1 nAChR cDNA (pRBG4-vector) was created using mutagenic primers 5′-CTATGACAGCTCTGTGGTGGC-3′ and 5′-CACAGAGCTGTCATAGGTCCAG-3′, and Phusion Hot Start DNA-polymerase (New England Biolabs, Ipswich, MA, USA). Polymerase chain reaction was performed at the following conditions: 98 °C for 1 min, 25 cycles of 98 °C for 10–30 s, 55–70 °C for 1 min, 72 °C for 3–4 min, and extended at 72 °C for 7 min. Then the PCR products were digested with the DpnI restriction enzyme (New England Biolabs) for elimination and the parental DNA and XL1-Blue competent cells (Evrogen) were transformed with the digested PCR products. The mutant mouse α1 nAChR cDNA was sequenced to confirm the mutation of interest (Evrogen).

*Isolation and purification of Makaluvamine G.* Makaluvamine G was isolated and purified from the Australian collection of *Zyzzya fuliginosa* as described in reference [39]. Earlier, this natural product was obtained from an Indonesian marine sponge *Histodermella* sp. [13].
